# Endovascular control of haemorrhagic urological emergencies: an observational study

**DOI:** 10.1186/1471-2490-6-27

**Published:** 2006-09-28

**Authors:** Bhaskar K Somani, Ghulam Nabi, Peter Thorpe, Sam McClinton

**Affiliations:** 1 Department of Urology, Aberdeen Royal Infirmary Hospital, Grampian NHS Trust, Aberdeen, Scotland, UK; 2 Department of Radiology, Aberdeen Royal Infirmary Hospital, Grampian NHS Trust, Aberdeen, Scotland, UK

## Abstract

**Background:**

Transarterial embolisation (TAE) is an effective method in control of haemorrhage irrespective of the nature of urological emergency. As the technique and technology have evolved, it is now possible to perform highly selective embolisation. The aim of this study was to critically appraise feasibility and efficacy of therapeutic TAE in control of haemorrhagic urological emergencies using selective and non-selective embolisation. Specifically, we aimed to assess the impact of timing of embolisation on the requirement of blood transfusion and long-term morphological and functional follow-up of embolised organs.

**Methods:**

This is a single institutional observational study carried out between March 1992 and March 2006. Records of all patients who underwent selective and non-selective angioembolisation to control bleeding in urological emergencies were reviewed. Data on success rate, periprocedural complications, timing of embolisation, requirement of blood transfusion and the long-term morphological and functional outcomes of embolised organs was recorded.

**Results:**

Fourteen patients underwent endovascular control of bleeding as a result of trauma, iatrogenic injury and spontaneous perinephric haemorrhage during a period of 14 years. All these patients would have required emergency open surgery without the option of embolisation procedure. The mean time between the first presentation and embolisation was 22 hours (range 30 minutes to 60 hours). Mean pre-embolisation transfusion requirement was 6.8 units (range 0–22 units). None of the patients with successful embolisation required post-procedural blood transfusion. Permanent haemostasis was achieved in all but one patient, who required emergency nephrectomy. There were no serious procedure related post-embolisation complications.

**Conclusion:**

Endovascular control using transarterial angioembolisation is an effective method for managing haematuria or haemorrhage in urological emergencies. Wherever and whenever indicated, this option should be considered early in the management of these cases.

## Background

Transarterial angioembolisation (TAE) is a well-established endovascular treatment of life threatening haemorrhagic urological emergencies [1]. Massive bleeding causing heamodynamic instability and requiring expeditious intervention can occur either during a surgical procedure or it could be a presenting symptom [2-5]. Such a clinical scenario is encountered in three clinical settings: haemorrhage following blunt or penetrating trauma, following iatrogenic injuries during a procedure or spontaneously with or without underlying pathology. The common underling renal pathologies are: arteriovenous malformations (AVM), renal angiomyolipoma (AML) and underlying renal cell carcinoma [1,6,7]. Control of bleeding can be achieved either by open surgical procedure or minimally invasive transarterial embolisation. Several case reports have reported advantages of TAE such as rapid recovery, short hospital stay and early resumption of physical activities [8]. However, there are several unanswered questions such as the timing of embolisation and its impact on the blood transfusion requirement and hospital stay, material for angioembolisation, complications, long-term morphological and functional follow up of embolised organs.

The objectives of present study are to assess the:

1. Feasibility and efficacy of transarterial embolisation in haemorrhagic urological emergencies.

2. Impact of timing of intervention on blood transfusion requirement.

3. Long term follow up of embolised organs.

## Methods

The records of all patients who underwent TAE procedures between March 1992 and March 2006 at a single centre (Aberdeen Royal Infirmary Hospital) were reviewed. The patients were identified from the hospital coding system and from a prospective radiological database using procedural codes. The hospital audit committee approved this study. All procedures were performed on an emergency (requiring immediate intervention) basis by experienced interventional radiologists in close collaboration with consultant urological surgeons. Data was extracted on pre-designed data extraction sheets. Following variables were recorded for each patient: age, sex, clinical presentation, presence of concurrent disease, blood pressure and heart rate, haemoglobin concentration, requirement of pre-embolisation blood transfusion, underlying pathology, timing of embolisation since their first presentation, embolisation agents, selective or non-selective embolisation, post-embolisation transfusion requirement and complications, hospital stay and long term outcome such as appearances of kidneys on imaging and blood pressure. The success of procedure was defined as complete occlusion of blood flow on post-embolisation angiography.

## Results

**Figure 1:**
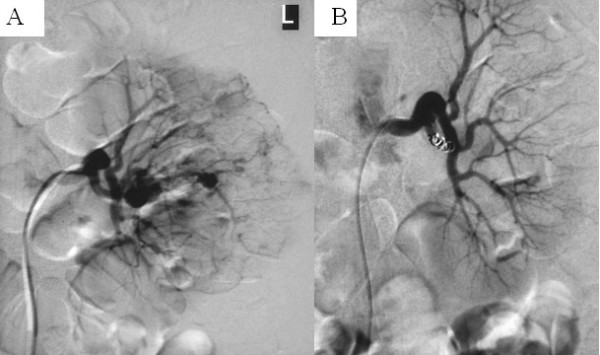
Angiography of spontaneous perirenal haemorrhage. A, Pre-embolisation. B, Post-embolisation of a branch of renal artery.

**Figure 2:**
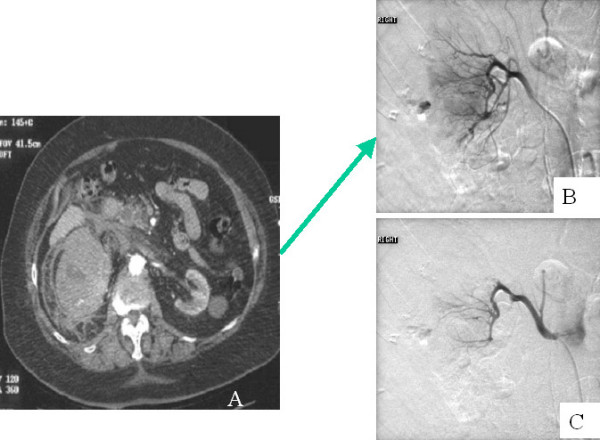
Spontaneous perirenal haemorrhage. A, Computerised tomography showing spontaneous perirenal haemorrhage. B, Angiography Pre-embolisation. C, Angiography Post-embolisation.

**Figure 3:**
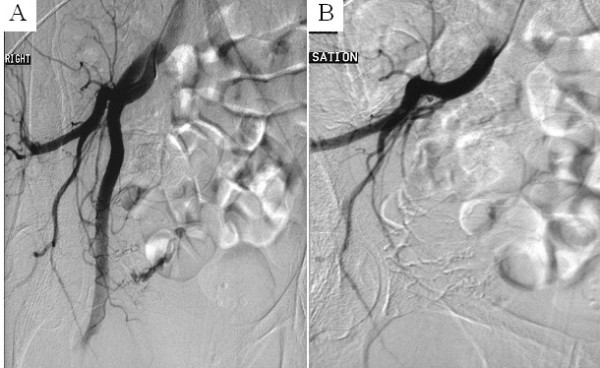
Angiography post transurethral resection of bladder tumour. A, Pre-embolisation. B, Post-embolisation of anterior division of internal iliac artery.

A total of 48 embolisation procedures were carried out in 40 urological patients between March 1992 and March 2006. Out of these, fourteen patients had TAE for control of haemorrhage as an emergency (Table 1). There were equal number of males and females. The mean age of these patients was 53.2 years (range 19 – 81 years). Various indications of embolisation are shown in Table 1. There were 4 non-selective and 10 selective embolisation procedures. The characteristic angiographic appearances of spontaneous perirenal haemorrhage and haemorrhage during a transurethral resection of bladder tumour are shown in Fig. 1A and 1B, 2A and 2B and 3A and 3B respectively.

Embolisation was successful in all except one patient (7%), who continued to have bleeding in post-embolisation period and required emergency nephrectomy after 48 hours. The histopathology showed renal cell carcinoma. The duration between the first presentation and embolisation was 22 hours (range 30 minutes to 3600 minutes). The mean pre-embolisation transfusion requirement was 6.8 units (range 0 to22 units). None of the patients any post-procedural blood transfusion. The embolisation agents included poly vinyl alcohol (PVC) particles (n = 2), coils (n = 11) and spongostan (n = 1). There were no major complications in our series. Minor complications in the form of post-embolisation syndrome (PES) were seen in seven patients [9]. These included fever (n = 2); pain (n = 3); nausea and vomiting (n = 1); and a combination of all three in one patient. All these were managed conservatively using analgesics, anti-pyretics and anti-emetics. The mean follow-up was 32 months (range 2 to – 156 months). All patients had follow-up functional imaging in form of a renogram or DMSA scan. They also had cross-sectional imaging in the form of USS or CT scan.

Patients with non-selective embolisation had small shrunken kidneys on follow up imaging. There were no morphological changes as seen on follow up imaging in the group of patients with selective embolisation. None of the patients developed new onset or deterioration in the control of hypertension.

## Discussion

Findings of the present study suggest that endovascular control of bleeding is a highly effective method, especially in emergency situations. Bleeding in all cases was intractable and would have required open surgical intervention to control haemorrhage, if embolisation facilities were not available or failed. Extravasation of contrast on a pre-embolisation angiography was seen in all the cases, suggesting an ongoing bleeding. There was a significant reduction in the requirement of blood transfusion following embolisation making us to conclude that this procedure should be offered much early in the course of management. The complications profile, especially minor and easily manageable re-emphasised the safety of this procedure.

Therapeutic transarterial angioembolisation (TAE) has been used successfully for managing a variety of benign and malignant urological conditions for the past 30 years. The use of this technique in controlling massive bleeding in urological injuries could be life saving [10]. Renal artery or injury to its branch following trauma or during percutaneous procedure for stone removal can be accurately diagnosed using angiography and treated by percutaneous embolisation techniques. Phadke et al [4] reported treating iatrogenic renovascular injury in 27 patients after percutaneous renal procedure. A superselective embolisation technique and early intervention for good results is generally recommended [5] mentioned a posterolateral initial puncture done with a bull's eye technique for PCNL's. They found a positive correlation of the stone size with occurrence of these injuries attributing to a greater manoeuvring with larger stones. Kessaris et al [2] performed 2,200 percutaneous renal procedures with 17 (0.8%) patients requiring angiography and embolisation for significant bleeding. Only 2 patients required open exploration following embolisation. In our experience of 1,800 PCNL's during the period of this study, 5 patients (0.3%) had iatrogenic vascular injury and needed embolisation. None of the cases required open surgery.

Spontaneous perirenal haemorrhage is an emergency where an early and accurate diagnosis is the key to avoid unnecessary nephrectomy. In an acute setting embolisation may be necessary to stop the haemorrhage [11]. A recent meta-analysis shows that nephrectomy and partial nephrectomy remain the commonest (70–75%) surgical interventions for perirenal haemorrhage [12]. We managed both cases of spontaneous perirenal haemorrhage by selective embolisation with a good outcome.

Takebayashi et al [7] reported their experience of TAE in bleeding renal AV malformation with a good long-term outcome. All patients except one had preserved renal function on follow up. The mean area of renal infarction in their series was 15.7 +/- 6.9%. The excellent functional results reported in this study were attributed to precise pre-embolisation calculation of contrast material for a particular target and frequent checks on the residual flow in the vessels to prevent reflux of contrast agent.

Kothary and colleagues [13] reported angioembolisation for control of angiomyolipoma (AML) in 30 patients. A high risk of recurrence of bleeding was reported in patients with associated features of tuberous sclerosis (TS). This subgroup of patients would need lifelong imaging surveillance repeat embolisation, if required.

Nabi et al [14] reported successful palliative management of intractable haematuria due to bladder tumour by embolisation of anterior division of internal iliac artery in 6 patients with no recurrence at a mean follow up of 22-month. Gujral et al [15] suggested an extended role of this procedure in peri-operative phase to control intractable bleeding following transurethral resection of bladder tumour. One patient in present study required embolisation in immediate postoperative period following transurethral resection of bladder tumour. This patient had a large bladder tumour and complete resection was not possible in a single session. Our experience suggests that this option must be seriously considered patients with post-procedural bleeding, where endoscopic control is not possible or difficult to achieve.

Although, this study has reiterated the feasibility and effectiveness of angioembolisation procedure in haemorrhagic urological emergencies especially significant reduction in blood transfusion requirement, there are several limitations worth consideration such as:

• This is a small case series of patients managed at a tertiary care centre. The applicability of findings from this study may not be applicable to small centres or where these facilities are not available.

• The long-term follow up of four patients with non-selective embolisation showed small contracted kidneys. All of these patients were asymptomatic with no recorded hypertension at their last follow up. However, large studies with long-term follow up are necessary to address the issue of hypertension in small contracted kidneys following angioembolisation. This is important in view of reversibility of hypertension following surgical intervention [16,17].

## Conclusion

Endovascular control using trans arterial angioembolisation is an effective method for managing haematuria or haemorrhage in urological emergencies. Wherever and whenever indicated, this option should be considered early in the management of these cases.

## Competing interests

The author(s) declare that they have no competing interests.

## Authors' contributions

BS made substantial contribution to the collection of the data and drafted the manuscript. GN made substantial contribution to the design, revised the manuscript for important intellectual content. PT assisted in the preparation of manuscript. SM conceived the study, and participated in its design and coordination. All authors read and approved the final manuscript.

## Pre-publication history

The pre-publication history for this paper can be accessed here:



## Figures and Tables

**Table 1:** Summarises the experience of transarterial angioembolisation (TAE) in Urological Emergencies

	**Age/Sex**	**Presentation (No. of blood units transfused pre/post embolisation)**	**Indication (Primary pathology)**	**Outcome**	**Follow-up**
1	32/F	Haematuria, fainting (3/0)	Angiomyo-lipoma	Successful.	2 months
2	74/F	Iatrogenic -Haematuria following TURBT (9/0)	Iatrogenic – TURBT	Successful.	1 year
3	59/M	Flank pain, Unstable patient (8/0)	Spontaneous perirenal haemorrhage	Successful.	1 year
4	76/M	On-table bleeding (0/0)	Iatrogenic- renal biopsy	Successful	1 year
5	48/M	On-table bleeding (0/0)	Iatrogenic- renal biopsy	Successful	6 months
6	54/F	Haematuria, Rt loin pain (8/0)	AVM (Arterio-venous malformation)	Successful	2 years
7	19/M	RTA, Retroperitoneal haematoma (15/0)	Rt adrenal gland haemorrhage	Successful	3 months
8	47/F	On-table bleeding (PCNL)(2/0)	Iatrogenic – Injury to aberrant Lt renal art	Successful	2 years
9	74/M	On-table bleeding (PCNL)(4/0)	Iatrtogenic – Injury to segmental renal art	Successful	6 months
10	43/F	3 days post PCNL (2/0)	Iatrogenic – Traumatic AV fistula	Successful	13 years
11	21/M	5 days post PCNL (22/0)	Iatrogenic – Traumatic AV fistula	Successful	6 months
12	45/M	13 days post PCNL (2/0)	Iatrogenic – Traumatic AV fistula	Successful	3 years
13	72/F	RTA, Abdo pain, Retroperitoneal haematoma (15/14)	Co-incidental Lt renal tumour	Unsuccessful nephrectomy after 48 hours	7 years
14	81/F	Haematuria, Rt loin pain (6/0)	Spontaneous perirenal haemorrhage	Successful	4 months

(TURBT – transurethral resection of bladder tumour, Rt – right, RTA – road traffic accident, Abdo – abdominal, Lt – left, AV – arteriovenous, PCNL – Percutaneous nephrolithotomy)
